# Effectiveness of therapeutic strategies for patients with neck pain: Protocol for a systematic review and network meta-analysis

**DOI:** 10.1097/MD.0000000000014890

**Published:** 2019-03-15

**Authors:** Qiang Gao, Weipeng Gao, Qing Xia, Chunyu Xie, Jian Ma, Liangzhen Xie

**Affiliations:** a Taian City Central Hospital, Taian; b Heilongjiang University of Chinese Medicine; c First Affiliated Hospital, Heilongjiang University of Chinese Medicine, Harbin, China.

**Keywords:** neck pain, network meta-analysis, protocol, therapeutic strategy

## Abstract

**Background:**

Neck pain is a common discomfort or more intense forms of pain in the cervical region. Neck pain has a large impact on individuals and their families, communities, healthcare systems, and businesses throughout the world. Therapeutic strategies are widely used for patients with neck pain in clinical practice, but the effectiveness of each therapeutic strategy is still unclear. The aim of this study is to assess the efficacy and safety of therapeutic strategies for neck pain.

**Method:**

Seven electronic databases will be searched regardless of publication date or language. Randomized controlled trials will be included if they recruited participants with neck pain for assessing the effect of each therapy. Primary outcomes will include pain score. The risk of bias will be assessed by 2 authors using the Cochrane tool of risk of bias. Network meta-analysis in random effects model will be conducted to estimate the indirect and mixed effects of therapeutic strategies for neck pain by R-3.5.1 software. The confidence in cumulative evidence will be assessed by grading of recommendations assessment, development, and evaluation.

**Results:**

This study will be to assess the effect and safety of therapeutic strategies for neck pain.

**Conclusions:**

This study will assess the effect of different therapeutic strategies for neck pain and provide reliable evidence for the choice of treatments.

**Systematic review registration:**

PROSPERO (CRD42019102385).

## 1 Introduction

Neck pain (NP) defined as discomfort or more intense forms of pain in the cervical region, is common, disabled and costly throughout the world. NP and its disability associated with NP have a large impact on individuals and their families, communities, healthcare systems, and businesses. NP has become the fourth leading cause of disability and more than 80% of individuals are affected.^[1,2]^ From a 2010 survey in American, about 10.2 million individuals visited the physician offices and hospitals for NP every year.^[3]^ A higher prevalence was found in officers, computer workers, women, and middle-aged people.^[4]^ In terms of global burden,^[5]^ NP was ranked as the 21st, whose DALYs added up to 33.6 M in 2010. Thus, NP is still a common and serious public health problem, and effective therapeutic strategies are required for the prevention.

The safety and effective method to relieve pain is important for individuals who suffer from long-term NP. Several therapeutic strategies are used to alleviate NP and its associated disorders, including conservative therapy, alternative, and complementary medicine treatments (acupuncture, massage, traction, Yoga and exercise, etc), injection, and surgery.^[1,6]^ Additionally, the evidence showed that the differential effectiveness of many therapeutic strategies for NP were based on subpopulations (such as age, sex, race, and stage) and therapeutic dosage was in terms of duration, intensity, and frequency.

To our knowledge, various therapeutic strategies were widely used for patients with NP in clinical practice, but the effectiveness of each therapeutic strategy was still unclear.^[1,6]^ Although some meta-analyses had been published,^[7–12]^ there was a lack of multiple comparisons in clinical studies so that it was more difficult to make the ideal choice. Network meta-analysis (NMA) was a way to assess the effects of more than 2 treatments for the same condition by direct and indirect comparisons.^[13]^ Therefore, we conducted the first NMA that comprehensively integrated the eligible randomized controlled trials (RCTs). Meanwhile, we intended to assess the effect of different therapeutic strategies for NP and provide reliable evidence for the choice of treatments.

## 2 Methods

### 2.1 Objectives and registration

This review will be to assess the efficacy and safety of therapeutic strategies for patients with NP. This review protocol has been registered in the PROSPERO that is the International Prospective Register of Systematic Reviews (https://www.crd.york.ac.uk/PROSPERO), and its registered number was CRD42019102385.

### 2.2 Inclusion criteria

#### 2.2.1 Types of studies

The inclusion criteria of studies will be RCTs in this systematic review regardless of publication status and language.

#### 2.2.2 Types of participants

In our study, participants will be diagnosed as NP regardless of their age, sex, or race.

#### 2.2.3 Types of interventions

In our study, we will evaluate the efficacy of every therapy for patients with NP in clinical practice.

#### 2.2.4 Types of outcome measures

In our study, primary outcomes will include pain score. Secondary outcomes will include neck function and disability, health-related quality of life, and adverse events.

### 2.3 Search methods for the identification of studies

We will search the 7 electronic databases regardless of publication date or language, including Cochrane Library, MEDLINE, EMBASE, Chinese BioMedical Database, China National Knowledge Infrastructure, Chinese VIP Information (VIP), and Wangfang Database. We will conduct different strategies for databases based on trial terms (random, trial, group), symptom terms (neck, cervical, pain, ache), treatment terms (manual therapy, massage, traction, mobilization, acupuncture, collar, Yoga and exercise, training, injection, surgery, corticosteroid, nonsteroidal anti-inflammatory drugs).

### 2.4 Data collection

**Figure 1:**
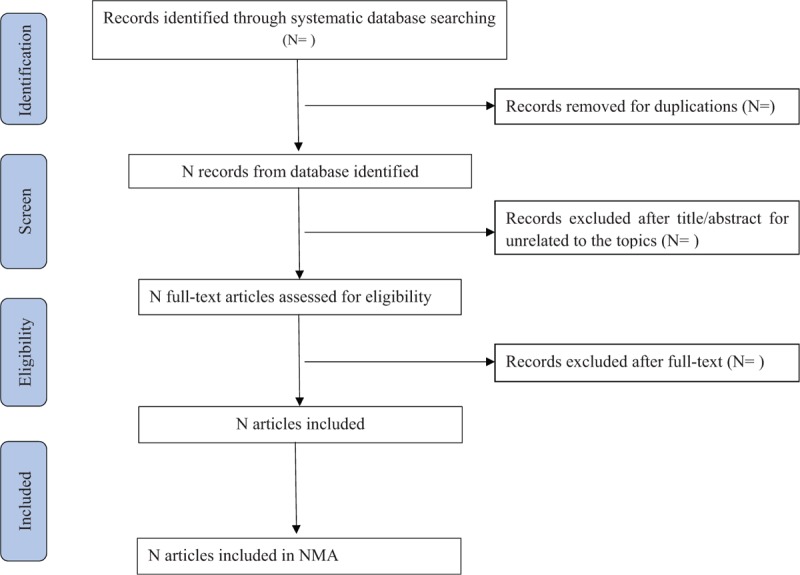
Flow chart of study selection.

Two reviewers (QG and WPG) will independently assess the titles and abstracts of all articles identified from electronic databases. Full-text articles will be scanned for all potentially relevant articles. If there is any disagreement on the selection of articles, they will be discussed with the third author (LZX). Two reviewers (QG and WPG) will independently extract the relevant information based on a standard data extraction table. Information will include baseline characteristics (publication of year, author, country, and sample size), participants (age and sex), intervention and control, outcomes. A PRISMA flow chart will be used to show the whole process (Fig. 1).

### 2.5 Assessment of the risk of bias

Two reviewers (QG and WPG) will independently assess the risk of bias by the Cochrane tool of risk of bias (V.5.1.0). The following items will be assessed: random sequence generation (selection bias), allocation concealment (selection bias), blinding (performance bias and detection bias), incomplete outcome data (attrition bias), selective outcome reporting (reporting bias), and other bias. The judgments of evaluated domains will include high, low and unclear. Disagreements will be resolved by discussion by arbiter (LZX).

### 2.6 Data synthesis and statistical analysis

#### 2.6.1 Intervention comparisons: direct

For continuous variables, mean difference or standardized mean difference with their 95% confidence intervals (CIs) will be reported. For categorical variables, risk ratios or odds ratio with their 95% CIs will be summarized. Standard pairwise meta-analysis in random effects model will be conducted by R-3.5.1 software where heterogeneity of interventions permit. For insufficient or missing data, we will contact the authors by e-mail or phone as much as possible.

#### 2.6.2 Intervention comparisons: indirect and mixed

NMA in random effects model will be conducted to estimate the indirect and mixed effects of therapeutic strategies for NP by package netmeta verison 1.13 of R-3.5.1 software. The inconsistency between direct and indirect comparisons was assessed by node-splitting method. The rank of each treatment for different outcomes will be summarized and evaluated by surface under the cumulative ranking curve.

#### 2.6.3 Subgroup and sensitivity analysis

Heterogeneity will be tested by standard Chi-squared statistic and Higgins *I*^2^ statistic. Considered of possible significant heterogeneity or inconsistency, subgroup analysis will be performed in order to explore the differences in age, sex, methodological quality, subtypes, and race/ethnicity. Additionally, if there are sufficient studies, sensitivity analysis will be performed in order to test the robustness of findings.

#### 2.6.4 Reporting biases

Funnel plots will be drawn to identify whether there will be the potential for small study bias if there are sufficient studies. If there are asymmetry of funnel plots that suggest possible small study effects, the results of analysis will be explained cautiously.^[14,15]^

#### 2.6.5 Confidence in cumulative evidence

Based on the grading of recommendations assessment, development, and evaluation, the level of evidence on outcomes will be assessed. The quality of the body of evidence will be assessed based on 5 factors, including study limitations, effect consistency, imprecision, indirectness, and publication bias. The assessments will be categorized as high, moderate, low, and very low quality.

## 3 Ethics and dissemination

Ethical approval is not appropriate, on account of this protocol for NMA. In our study, there will be no patients recruited, and no data gathered from patients. This review will be disseminated by the approach of peer-reviewed publications.

## Author contributions

LZX and JM developed the study protocol. QG and WPG developed the search strategy with the supervision of LZX. QG and WPG will scan the included studies, extract the data and assess the risk of bias. QG and QX will perform data analysis with the supervision of LZX and JM. All authors (QG, WPG, QX, CYX, JM, and LZX) will contribute to data interpretation. QG, WPG, QX, CYX, JM, and LZX drafted and revised the manuscript. All authors have read and approved the final version of the manuscript.

**Conceptualization:** Jian Ma, Liangzhen Xie.

**Data curation:** Qiang Gao, Weipeng Gao, Qing Xia.

**Formal analysis:** Qiang Gao.

**Funding acquisition:** Qiang Gao.

**Methodology:** Weipeng Gao, Qing Xia, Liangzhen Xie.

**Software:** Qiang Gao, Weipeng Gao, Qing Xia.

**Supervision:** Jian Ma, Liangzhen Xie.

**Writing – original draft:** Qiang Gao, Weipeng Gao, Qing Xia, Chunyu Xie, Jian Ma, Liangzhen Xie.

**Writing – review and editing:** Qiang Gao, Weipeng Gao, Qing Xia, Chunyu Xie, Jian Ma, Liangzhen Xie.
